# Assessing Niche Shifts and Conservatism by Comparing the Native and Post-Invasion Niches of Major Forest Invasive Species

**DOI:** 10.3390/insects11080479

**Published:** 2020-07-29

**Authors:** Vivek Srivastava, Wanwan Liang, Melody A. Keena, Amanda D. Roe, Richard C. Hamelin, Verena C. Griess

**Affiliations:** 1 Department of Forest Resources Management, The University of British Columbia, Vancouver, BC V6T 1Z4, Canada; verena.griess@ubc.ca; 2 Center for Geospatial Analytics, North Carolina State University, Raleigh, NC 27695, USA; wliang5@ncsu.edu; 3 Northern Research Station, USDA Forest Service, Hamden, CT 06514, USA; melody.keena@usda.gov; 4 Great Lakes Forestry Center, Natural Resources Canada, Sault Ste. Marie, ON P6A 2E5, Canada; amanda.roe@canada.ca; 5 Department of Forest and Conservation Sciences, The University of British Columbia, Vancouver, BC V6T 1Z4, Canada; richard.hamelin@ubc.ca

**Keywords:** niche conservatism, niche shift, invasive species, *Anoplophora glabripennis*, *Sirex noctilio*, *Ophiostoma ulmi*, *Ophiostoma novo-ulmi*, *Phytophthora ramorum*

## Abstract

Invasive species experience biotic and abiotic conditions that may (or may not) resemble their native environment. We explored the methodology of determining climatic niches and compared the native and post-invasion niches of four invasive forest pests to determine if these species experienced shifts or changes in their new climatic niches. We used environmental principle components analysis (PCA-env) method to quantify climatic niche shifts, expansions, and temporal changes. Furthermore, we assessed the effect of variable selection in the delineation and comparison of niche space. We found that variable selection influenced the delineation and overlap of each niche, whereas the subset of climatic variables selected from the first two PCA-env axes explained more variance in environmental conditions than the complete set of climatic variables for all four species. Most focal species showed climatic niche shifts in their invasive range and had not yet fully occupied the available niche within the invaded range. Our species varied the proportion of niche overlap between the native and invasive ranges. By comparing native and invasive niches, we can help predict a species’ potential range expansion and invasion potential. Our results can guide monitoring and help inform management of these and other invasive species.

## 1. Introduction

The geographic range of a species results from the complex interaction of many biotic and abiotic factors [1,2]. Climatic conditions are considered to be major determinants of a species range [3]. However, climate alone cannot always predict the distribution of a species, as many species do not occupy all available habitats due to accessibility. Geographic barriers such as mountain ranges and oceans limited the migration of many species to new areas. With increased trade, anthropogenic movement of alien species has accelerated in the past century breaching these historic barriers and permitting the unprecedented movement of organisms around the globe [4]. As alien species are introduced to novel habitats, they are exposed to a variety of abiotic and biotic conditions that may (or may not) resemble their native range. Climate along with other range-limiting factors plays a key role in the future outcomes of these introductions, once human mediated dispersal and/or elimination of dispersal boundaries allows movement beyond the native range [5,6].

Ecological niche can be defined as a multi-dimensional environmental space within which a species can maintain its source populations [7]. The ecological niche can be further described as fundamental and realized niche. The fundamental niche, which is determined by the physiology of the species (such as heat-tolerance), represents an abiotic environmental space where a species can thrive [1,7]. Realized niche represents a portion of fundamental niche where species can actually thrive after considering biotic factors such as competition, predation and mutualism [1,7]. Predicting the potential distribution of invasive species has received increased attention [8]. Species distribution models (SDMs) have become a popular tool to investigate and predict the potential distribution of a new invader [9]. SDMs rely on matching environmental conditions available in species native and invasive range to predict the potentially suitable habitats of invasive species in the new range. As a subset of fundamental niche, climatic niche only focuses on the climate-related abiotic factors. In order to gain understanding on fundamental niche, information on species functional characteristics and physiological thresholds is required. Recent improvements in SDMs with increased availability of species occurrences and climatic data have led to a wider use for investigating species climatic niche evolution during an invasion [6,10]. 

However, using SDMs to predict invasive niches has recently come under scrutiny. SDMs are based on the assumption of niche conservatism—that species occupy similar niches in both their native and invasive ranges—which is supported by little evidence [1,6]. Moreover, niche overlaps measured using SDMs are likely to vary depending on the extent and distribution of environmental gradients in the study area and potentially due to varying statistical assumptions and procedures related to model fitting [11]. 

Furthermore, invasive species may undergo evolutionary niche shifts through genetic drift, selection or hybridization that can modify their environmental requirements to match the available conditions in invasive ranges [3]. Given this, SDMs will not be able to precisely predict the potential range of an invasive species or the characteristics of the niche for invasive range distributions [8]. Moreover, SDMs are primarily based upon species occurrence records in their native range to describe a new potential distribution and may highly underestimate where an invasive species could survive. SDMs based on native ranges would also fail due to their inability to predict the post introduction influences of adaptations, interactions and dispersal barriers within the invasive range [12]. Given these constraints, doubts can arise whether the calculated niche corresponds to the one occupied by the given invasive; hence, leading to significant levels of uncertainty regarding the outcomes of the assessed niche shifts [13]. Thus, exploring niche shifts between native and invasive ranges may offer additional insights that might be useful in understanding range expansion and invasion potential of invading species [14]. 

To address the criticisms leveled at SDMs for predicting niche space, the ordination method proposed by Broennimann et al. [11] allows a direct comparison of the species environmental relationships within the environmental space and employs various maximization criteria to construct two dimensional representations of the niches using the associated environmental variables [11,15]. In addition, this method equally weighs all environmental variables and considers both species’ geographic and environmental spaces. Moreover, the approach corrects the densities of known species occurrences by considering the available environmental space and correcting for sampling biases. 

We used the ordination method [11] to evaluate and contrast post invasion climatic niche shifts across diverse populations of two insects (*Sirex woodwasp* (WW), *Sirex noctilio* (Fabricus); Asian longhorned beetle (ALB), *Anoplophora glabripennis* (Motschulsky)) and two pathogens (sudden oak death (SOD), *Phytophthora ramorum* (Werres), and Dutch elm disease (DED), *Ophiostoma ulmi* (Buisman) Melin and Nannf. and *O. novo-ulmi* Brasier), which are currently occupying new ranges. We also analyzed the effects of differences in choice of variables (all versus selected) in evaluating climatic niche features for these representative invasives.

*Sirex noctilio* is a wood wasp that is native to Europe, northern Asia and northern Africa where it has few negative impacts on Pinus plantations and rarely outbreaks [16,17]. The first invasive population of *Sirex noctilio* was found in New Zealand in the early 1900′s and it has since invaded many pine producing countries in the southern hemisphere (Australia, Uruguay, Argentina, Brazil, Chile, Western Cape, Eastern Cape, Kwazulu-Natal, Mpumalanga and Limpopo, [18]). In 2004, it was first discovered in North America [19], and it is now found in seven states and two provinces. Genetic analyses have revealed a complicated invasion history, with invasive populations showing at least some level of admixture, meaning derived from more than one source population [18]. The Oceania populations came from Europe, then insects from there spread to South Africa and South America. South African populations also received insects from South America and an unknown location. South American populations received insects from Europe directly and Chile also received insects from the same unknown population as is found in South Africa. North American populations are a mixture of insects from South America, Europe and possibly Australia. Interestingly, the population of *Sirex* sampled in Switzerland shows invasion by a mixture of the unknown source and South American genotypes, even though it is part of the native range. It uses many *Pinus* species, but *Pinus radiata*, *Pinus taeda* and *Pinus patula* are very susceptible to attack. In the invaded areas in the southern hemisphere damaging outbreaks occur. Tree death occurs after female wasps oviposit eggs along with a phytotoxic mucus and a symbiotic wood decay fungus (*Amylostereum areolatum*) into stressed trees. Since the larvae require the decaying wood to develop, conditions must be right for fungal growth to occur. The temperature range over which *Sirex* can complete development is 12.5–33.5 °C but 60% die at 33.5 °C [20]. In warmer climates it can have two generations per year and in colder climates it may take multiple years to complete a generation [21].

Asian longhorned beetle is a large cerambycid woodboring beetle that attacks >100 species of hardwood trees. ALB is native to Asia (21 to 45° N latitude), though predominantly found within central and northern China, as well as the Korean peninsula. ALB infested large monocultures of hybrid poplars and windbreaks of willows that were planted as part of an afforestation effort in northern China in the 1970s and 1980s, and subsequently became recognized as an important forest pest [22,23,24]. The first invasive population of ALB in North America was detected in New York in 1996 [25,26,27] and the first detection in Europe was in 2001 [28]. Subsequently more invasive populations have been discovered in North America between 38 and 44° N latitude (five states and one Canadian province) and Europe between 33 and 60° N latitude (11 countries, [26,27]). To date the most northern invasive population of ALB was in Finland and the most southern is in Lebanon, indicating that the species can tolerate a wide thermal range. The source of the invasive populations remains unclear due to complex genetic structure in the native range and potentially reticulated invasion pathways due to secondary spread from invasive populations [26,29]. 

Sudden oak death is an invasive disease caused by *Phytophthora ramorum*, a pathogen that was discovered simultaneously on oaks in California and rhododendron in Europe in the mid-1990s [30,31]. The pathogen is believed to be native to Indochina and the source of the two introductions was recently discovered to be in Vietnam [32]. It has an extremely broad host range, which facilitates its spread through the horticulture industry. However, the pathogen can also spread from nurseries into natural forests where it can cause severe outbreaks. The epidemic spread is quite different in nurseries, where it is driven by the movement of infected material via trade, and in forests where the presence of sporulating hosts, such as bay laurel and tanoak in California, is responsible for the production of spores and the intensification of outbreaks. In North America, the pathogen sporulation and host infection appear to be restricted to the rainy season in the Pacific Northwest and the annual variation in precipitation is likely to be an important factor influencing the epidemic [31]. The disease has so far been reported in western North America, throughout Europe and recently in Japan and Vietnam [30,32].

The Dutch elm disease is caused by a pathogen complex that comprises *Ophiostoma novo-ulmi*, *O. ulmi* and *Ophiostoma himal-ulmi*. The origin of the pathogen is still unclear but is assumed to be in the Himalayas [33]. The first disease pandemic was caused by *O. ulmi* in the 1940s in North America and Europe, followed by a second pandemic by the more virulent *O. novo-ulmi*. These pathogens are vectored by elm bark beetles that carry the fungus spores into the vascular system of the host. They can be spread both by long-term transport of elm wood and by the spread of the beetle. The pathogens appear to thrive under various environmental conditions. In North America it is found from the West Coast to the East Coast and from Saskatchewan to Texas. All North American and European elms are susceptible to various degrees, but several Asian elm species are resistant.

All four representative species pose significant threats to tree health in their invasive ranges [34,35] and pose continuing risk of spread to new areas [36]. Currently, there is information on preferred hosts, responses to temperature and other biological information but the specific niche characteristics in their respective introduced and invasive ranges have not been defined or compared. Providing such information is important in understanding their ecology leading to better early detection rates. In the following we examine if the introduced invasive native populations occupy similar environmental conditions in their invasive and native ranges. We also calculated how stable the niche is, determined if the species niche is expanding/evolving over time, and evaluated if the entire area defined by the niche has been invaded yet in each introduced range to better understand the niche dynamics of the selected species. We predict that *Sirex* populations will show shifts in their niches and some degree of evolution. There seem to be two primary groupings (European and unknown source) of the strains that have become invasive and they seem to respond to biocontrol differently. These groups may also have different responses to temperature. *Sirex* causes more damage in the southern hemisphere than in the northern hemisphere, which may have multiple causes (hosts, biological controls, and climate). The introduced ALB populations will show shift in their niches possibly due to alterations in their host preferences and variations in climatic profiles, given the more northern populations in Europe and that the invaded range does not go as far south in latitude as it does in China. In addition, infested areas may represent a small part of potentially suitable areas. Similarly, SOD populations are very likely to show shifts in their niches due to shifts in their hosts and may expand their niche in the invasive ranges. The SOD pathogen is known to have a broad host range, but host jump can result in new outbreaks, as the outbreak on larch in Europe. The other big driver could be climate, as climate change could turn current inhospitable climates into disease-conducive climates. DED populations could show niche evolution due to expansion of the host and climate change. Moreover, some DED vectors are more efficient transmitters of the pathogen and can intensify outbreaks.

## 2. Materials and Methods 

### 2.1. Occurrence Data

**Figure 1:**
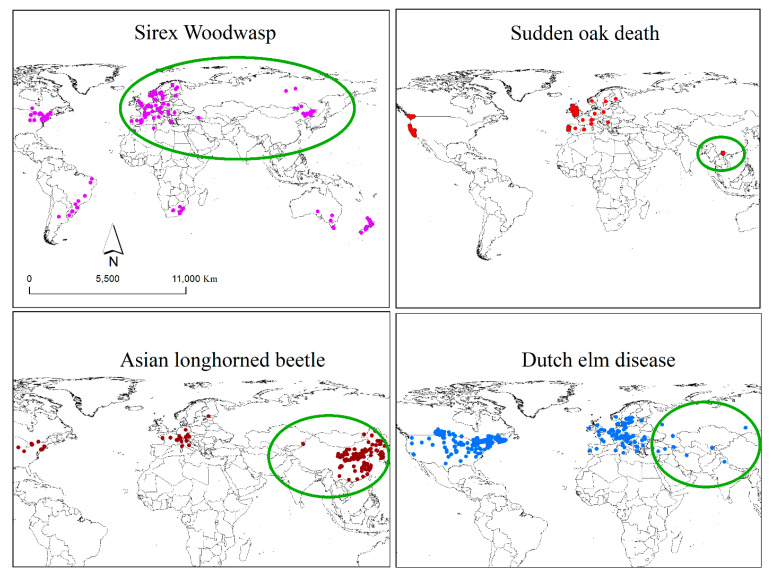
Geographical distribution of *Sirex* *Woodwasp* (WW), Asian longhorned beetle (ALB), sudden oak death (SOD), and Dutch elm disease (DED). Native ranges for each invasive are shown in green. Since the origin of DED is not known we assume them to be native to Asia for the purpose of comparison.

We compiled an occurrence database of WW, ALB, SOD, and DED from various sources, including, (1) records provided by the Canadian Food Inspection Agency (CFIA); (2) Global Biodiversity Information Facility database, an online database for species occurrences; (3) Centre for Agriculture and Bioscience International (CABI) invasive species compendium; and (4) scientific articles and maps (Figure 1) [32,37,38,39]. We used Google Earth (Google Inc 2020, Mountain View, CA, USA) to obtain proxy coordinates for records lacking geographic coordinates. In order to generate geographically unique occurrences and account for potential sampling bias we applied a buffer of 5 km around each record using spThin R package [40]. Considering dispersion abilities of the representative species a minimum convex polygon around the occurrences with an added dispersion distance of 1 degree was applied to define their geographic background in their respective regions. Following backgrounds were considered for each invasive: (1) WW—Eurasia (native, *n* = 116), North America (NA, *n* = 12), South America (SA, *n* = 12), South Africa (SF, *n* = 11), and Oceania (Australia + New Zealand, *n* = 33); (2) ALB—Asia (native, *n* = 149), Europe (EU, *n* = 23), and North America (NA, *n* = 26); (3) SOD—Indochina (native, *n* = 8), North America (native, *n* = 45), and Europe (EU, *n* = 53); and (4) DED—Asia (native, *n* = 10), Europe (EU, *n* = 105), and North America (NA, *n* = 209). It should be noted that the native range of SOD has been recently discovered and the presumed native range of DED has been poorly sampled hence we have limited data for these two species in their respective native ranges.

### 2.2. Climate Data

We obtained a complete set of 19 bioclimatic variables from the WorldClim database version 1.4 (http://www.worldclim.org/) [41], averaged for the 1950–2000 period at a spatial resolution of 2.5 arc minutes and masked them to match the extent of each species distribution. Following Broennimann et al. [11], we evaluated niche features of the focal species among their invasive ranges after calibrating them on their respective geographical regions. The selection of the relevant set of variables for each of the representative species was made using the R package “Maxent Variable Selection’’ [42]. The variables were selected in a progressive manner taking various regularization multipliers, variable contribution and autocorrelation into account. The final set of variables (selected set) were obtained from the model having the lowest Akaike’s Information Criterion for small sample size scores (AICc) [43]. The selected set of variables for WW and ALB included five variables, for SOD four and seven for DED.

### 2.3. Measuring Climatic Niche Shifts

We used the environmental principle components analysis (PCA-env) method proposed by Broennimann et al. [11] to test for shifts in the climatic niches of focal species after their introduction into their invasive ranges. This method allowed us to compare the environment conditions available to a species within a region with its observed locations and calculate the available environment space which was defined by the first two axes from the PCA. This method corrects for potential sampling biases by considering available environmental space within the entire background by applying a kernel smoothing function to occurrence densities. 

We compared the climatic conditions available for each species within their invasive ranges to those found within the native range. Our approach followed Silva et al. [44] where they created occurrence density models while correcting for the available environmental conditions for the studied species and then quantified niche overlap using Schoener’s D index [45], which varies from 0 (no niche overlap) to 1 (when the niches are identical). We then used this metric to test for niche equivalency and similarity. The niche equivalency test compares the observed niche overlap with the estimated overlap when occurrences are randomly reassigned to both backgrounds. The species occurrences were randomized in both backgrounds and Schoener’s D was recalculated 100 times to produce a null distribution of overlap scores which was then compared with the observed value [46]. The niche equivalency test used exact locations of species and did not take into account the entire available space, whereas the niche similarity test considered differences in the surrounding environment conditions available across the species distributional area [47]. We used the niche similarity test to assess niche shifts and conservatism. For detailed information on niche similarity test readers are referred to [11]. The similarity test examined if the observed overlap between the compared niches is different from the overlap between the observed niche in one range and randomly selected niches in the other range. A significant higher overlap score indicates more similar environmental conditions between two compared ranges than expected by chance [44]. The rejection of niche similarity hypothesis signifies that the environmental conditions occupied by the species in the invasive range are more similar to the environmental conditions occupied in the native range than would be expected by chance (overlap between native and invasive niche is larger than random expectation) [48]. Hence, a p-value less than 0.05 indicates niches that are more similar than expected by chance, while conversely a value greater than 0.05 signifies niches that are less similar than expected by chance [49,50]. Statistical significance of the niche similarity test was assessed on the basis of 100 randomizations and an alpha of 0.05. 

In order to provide more insight into the niche dynamics of the representative species we also calculated how stable the niche was, determined if the species niche is expanding/evolving over time, and evaluated if the entire area defined by the niche has been invaded yet. Niche stability and expansion (=1-niche stability) measure the proportion of occupied environmental space in the introduced range that is overlapping and non-overlapping, respectively, to that of the native range. To determine if the nonnative niche was completely invaded the proportion of native range that does not overlap with the invasive range was calculated. We used the package ecospat [51] in R [52] to obtain the proportion of climatic niche in each comparison as proposed by Guisan et al. [5]. The R code for the PCA-env was modified from Broennimann et al. [11] and Silva et al. [44] to perform the analysis. To assess the impact of variable selection on the niche analysis, we compared the results obtained using the complete set of climatic variables (n = 19) to the selected set of climatic variables. We discussed variations in the climatic niches of the focal species in the direction 1→2 (only considering native versus invasive ranges).

## 3. Results

**Figure 2:**
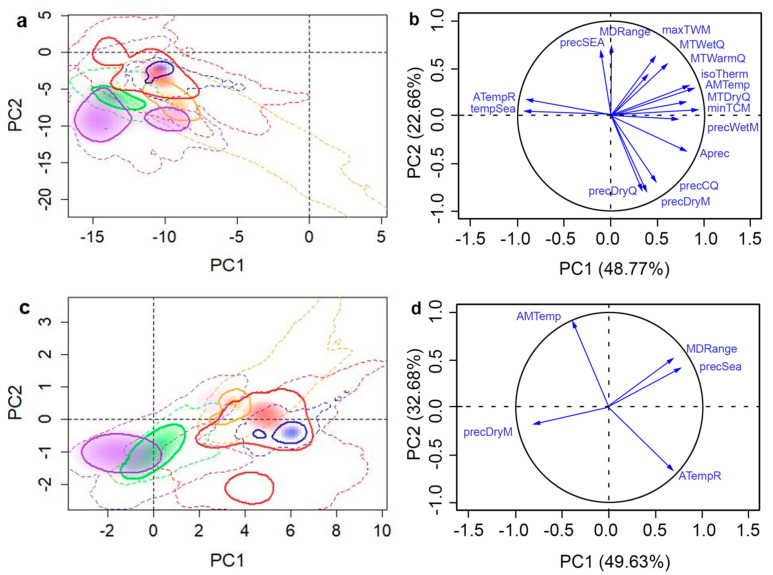
Native and invasive climatic niches of *Sirex* *Woodwasp* in different regions along with variable factor maps. Multivariate climatic space was calculated using the environmental principle components analysis (PCA-env) method. Results using all variables are shown in parts (**a**) and (**b**), whereas parts (**c**) and (**d**) shows the results obtained with selected variables. The solid and dashed lines delineate the niche occupied by the 50% densest population and all available climatic niche, respectively. Shadings correspond to the density of occurrences in each of the following regions: Africa (green, non-native), Oceania (orange, non-native), North America (blue, non-native), South America (purple, non-native), and Eurasia (red, native), respectively. The variance explained by the PC1 and PC2 is 71.43% for all variables and 82.31% for selected variables.

**Figure 3:**
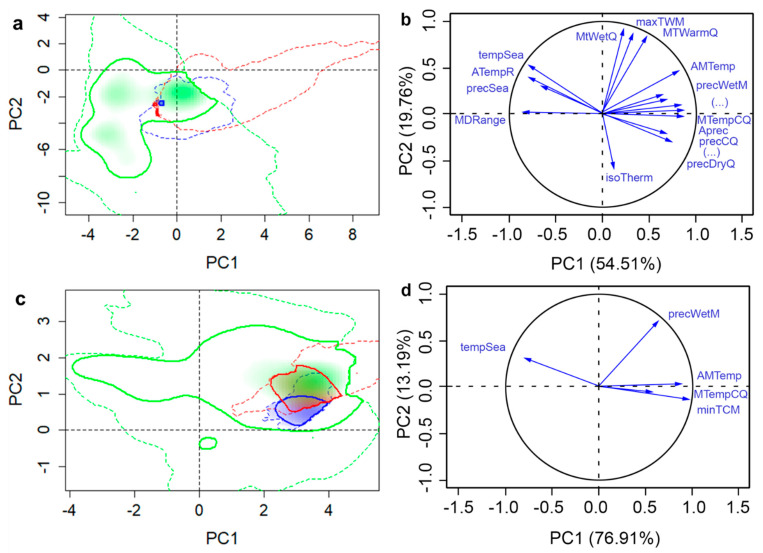
Native and invasive climatic niches of ALB in different regions along with variable factor maps. Multivariate climatic space was calculated using the PCA-env method. Results using all variables are shown in parts (**a**) and (**b**), whereas parts (**c**) and (**d**) shows the results obtained with selected variables. The solid and dashed lines delineate the niche occupied by the 50% densest population and all available climatic niche, respectively. Shadings correspond to the density of occurrences in each region: Asia (green, native), Europe (red, non-native), and North America (blue, non-native), respectively. The variance explained by the PC1 and PC2 is 74.27% for all variables and 90.10% for selected variables.

**Figure 4:**
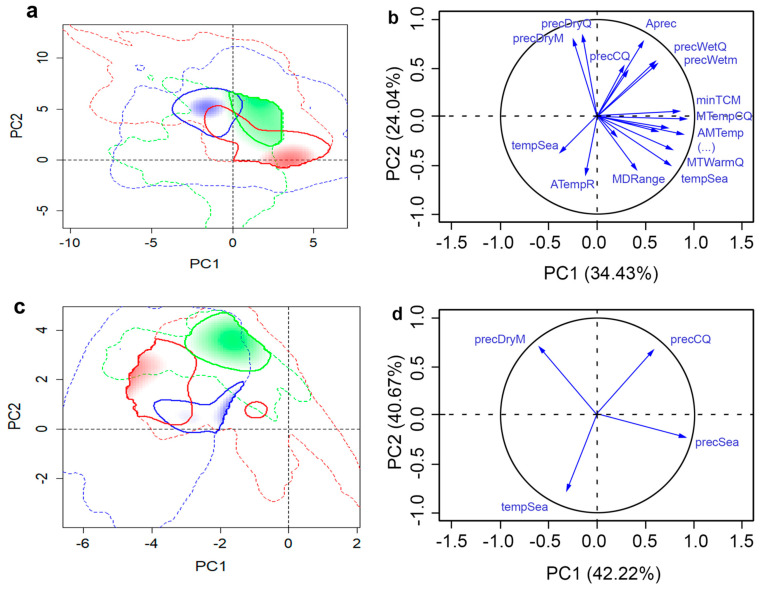
Native and invasive climatic niches of sudden oak death in Indochina (Vietnam), Europe, and North America along with variable factor maps. Multivariate climatic space was calculated using the PCA-env method. Results using all variables are shown in parts (**a**) and (**b**), whereas parts (**c**) and (**d**) shows the results obtained with selected variables. The solid and dashed lines delineate the niche occupied by the 50% densest population and all available climatic niche, respectively. Shadings correspond to the density of occurrences in each region: Europe (red, non-native), North America (blue, non-native), and Indochina (green, native), respectively. The variance explained by the PC1 and PC2 is 58.47% for all variables and 82.89% for selected variables.

**Figure 5:**
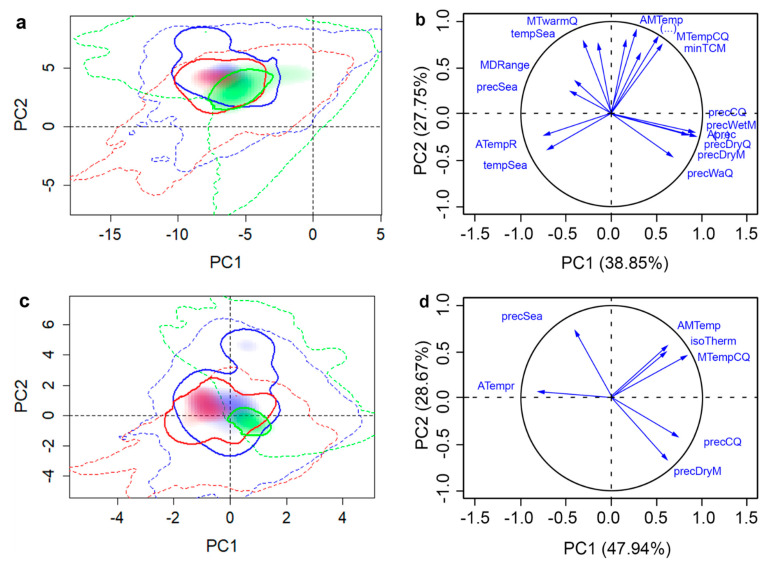
Native and invasive climatic niches of Dutch elm disease in different regions along with variable factor map. Multivariate climatic space was calculated using the PCA-env method. Results using all variables are shown in parts (**a**) and (**b**), whereas parts (**c**) and (**d**) shows the results obtained with selected variables. The solid and dashed lines delineate the niche occupied by the 50% densest population and all available climatic niche, respectively. Shadings correspond to the density of occurrences in each region: Asia (green, native), Europe (red, non-native), and North America (blue, non-native), respectively. The variance explained by the PC1 and PC2 is 66.60% for all variables and 76.61% for selected variables.

The selected set of variables for each species was substantially reduced from the original 19 climatic variables (Table 1). DED retained seven variables, while SOD retained four and the two insects had five within each of their sets. The combinations of selected set variables differed between each species (Table 1). The variance explained by the first two PCs by all variables and selected variables were 71.43% and 82.31% for WW, 74.27% and 90.10% for ALB, 58.47% and 82.89% for SOD, and 66.60% and 76.61% for DED, respectively (Figure 2, Figure 3, Figure 4 and Figure 5). The prominent increases (10.01–24.42%) of the total variance explained by first two PCs of selected variables can make the PCA-env method potentially incorporate more variance on variables that are critical to the fundamental niche than using all variables when evaluating niche similarities. Selection of important variables were further discussed in the Discussion section. When we compared these two datasets, selected vs. complete, we found that they produced different proportions of niche overlaps and other studied metrics, where the selected set of variables provided results that were closer to the observed species occurrences. Hereafter, we present only the results generated using the selected variable sets in direction (1→2) but provide details for each dataset in the provided tables (Table 2, Table 3, Table 4 and Table 5) for direct comparison. Additional results of niche comparison between introduced ranges versus native range (in direction 2→1) are provided in Appendix A (Table A1, Table A2, Table A3 and Table A4).

### 3.1. Sirex Woodwasp

All introduced populations of WW showed low niche overlap (0.01–0.19, Table 2) and were not similar to the native range (did not exhibit high similarity to the native range and showed variable niche overlap proportions that varied from 0.01 to 0.19 (Table 2). The overlap scores between the native and invasive ranges were very low for South America (0.01) and Africa (0.02). The WW populations in Africa and SA occupied more humid and colder environments than those found in the Australian and NA ranges. Whereas, the WW populations in Australia occupied warmer conditions compared to native and other invasive ranges of WW. Moreover, along with native populations, the Australian populations preferred drier environments (Figure 2). Climatic niches of the invasive ranges of WW showed moderate to low degrees of niche invasion when compared to the native range (0.57–0.91) indicating that there is considerable niche range into which the populations could expand. However, there was no expansion and stability observed in the niches of the Oceania and NA populations when compared to the native range. Populations in African and SA ranges showed moderate niche expansion and stability (Table 2). 

### 3.2. Asian Longhorned Beetle 

ALB has a large geographic distribution within its native range, spanning from 21 to 45° N degrees of latitude (Figure 1), with multiple invasions within North America and Europe. The ALB niches were defined by five climatic variables (bio 1, 4, 6, 11, and 13; Table 1), four of which are related to temperature. The niche overlap with the native distribution ranged from 0.17 to 0.33 for the invasive ranges in NA and EU respectively (Table 3), meaning that the NA niche showed less niche overlap with the native niche than EU due to greater overlap in occurrence density in the EU niche than NA (Figure 3c). NA populations exhibited a climatic niche that was significantly similar to the native range (Table 3). However, the introduced populations in the EU occupy locations that are warmer and more humid than those found in NA and the native range (Figure 3) and did not exhibit niche similarity to the native range (Table 3). The lack of significant similarity between native and EU invasive range shows that the populations of ALB in the EU range have different climatic preferences than observed in the native range. Despite this difference, climatic niches of all invasive ranges of ALB showed high degrees of niche filling, and little to no expansion and high niche stability when compared to the native range (Table 3). 

### 3.3. Sudden Oak Death

SOD has a broad geographic distribution in its invasive range in North America and Europe, but its native range was unknown until recently (Figure 1). The discovery of a very diverse population of the pathogen in South Asia makes that region a likely center of origin. Climatic niche analyses of SOD revealed different climatic preference of SOD in the invasive ranges as both introduced EU and NA populations had less similar niche to that of native range. We observed an overlap of only 0.9% in climatic niches of NA and native populations but 16% overlap between EU native populations (Table 4). The SOD populations in NA were exposed to more variations in temperature whereas those in EU were exposed to more variations in precipitation compared to the native range (Figure 4). Introduced populations occupied drier areas compared to the native range. Based on the complete set of climatic variables SOD populations in the invasive EU range occupied colder environments than populations in native and NA range (Figure 4). Most of the available SOD niche (99%) was not yet invaded in NA and 58.5% of the niche remained uninvaded in the EU range. A high degree (77.3%) of niche expansion was observed in the NA range whereas a moderate degree (58%) of niche expansion was observed in the EU range. Both invasive ranges had low niche stability (Table 4).

### 3.4. Dutch Elm Disease 

DED has a broad geographic distribution in its invasive range in North America and Europe. Its native range is believed to be Asia, where a close relative of the DED pathogen is found and is used here for the native range (Figure 1). DED niche overlap comparisons between the chosen “native” and invasive ranges also showed different overlap proportions ranging from 0.18 to 0.49 for invasive ranges in NA and EU, respectively. The DED populations occupied drier and warmer conditions in NA than in the EU range. The NA populations also showed a shift towards colder regions as compared to the ones in the EU range. Moreover, the populations in the native range were exposed to higher variation in precipitation than those found in the invasive ranges. However, invasive DED in both NA and EU occupied cooler environments than native populations. DED populations in all ranges preferred humid environments (Figure 5). Both EU and NA populations exhibited a climatic niche that was not significantly similar to the native range (Table 5). Climatic niches of the introduced ranges of DED showed highest degrees of niche filling when compared to the native range. However, expansion in the niches of EU (22%) and NA (57%) populations were observed. Furthermore, the EU range was found to be more stable than NA.

## 4. Discussion

In this study we demonstrate the importance of climatic variable selection when defining the climatic niches of invasive species [53]. When we used the more relevant variables for each species, we found different niche overlap values for each studied niche feature, compared with our results generated using all climatic variables. The results generated using a subset of variables produced results that were close to known distribution of our focal species. For e.g., results generated using select set of variables agrees with the fact that the DED populations in the presumed native range are distributed in areas having warmer conditions compared to the introduced ranges. Moreover, using the selected set it was shown that the NA populations had preferences towards colder areas. This finding matches with the known distribution of the pathogen where it is proven that *O. novo-ulmi* is better adapted to cooler climates than *O. ulmi*. On the contrary, when using all climatic variables we produced fairly opposite results to what we obtained with using selected set of variables. Our results suggest that using a complete set of bioclimatic variables for climatic niche analysis can include variables which are not relevant to the actual niche of respective species and further limit our understanding on accurate niche delineation. A few researchers have selected all 19 bioclimatic variables found in the WorldClim database [44], whereas several have chosen only variables considered relevant to assess the niche shift [54,55,56,57,58]. Here, based on our results, using a complete set of bioclimatic variables in niche mapping should not be recommended. Further, inappropriate selection of variables might not produce robust niche models and could produce false information on habitat quality and relative importance of variables in defining the niche [43,59]. Previously, other researchers also have demonstrated the importance of variable selection in determining suitable ranges for species establishment [43,60,61]. Title and Bemmels [62] found that including more variables that are likely to have direct relevance on ecological or physiological processes of target species can substantially improve the performance of niche models.

Selection of relevant factors when delineating the niches or predicting the potential distribution of invasive species [63,64] has been conducted using multiple approaches in practice. The commonly used approaches assess the correlation between variables and then choose a subset to avoid multicollinearity [65]. However, such method alone can fail to include biologically meaningful variables, which can further lead to misestimation of invasive species’ niche. Meanwhile, we are aware of the possibility that the method used in this research can lead to failing of including biological meaningful variables in the niche analysis. We strongly recommend using a subset of variables that are biologically important to the target species and have been obtained by a rigorous selection method for the niche analysis. When little physiological information is available for the target species to facilitate selection of biological meaningful variable, recently developed tools like kuenm [60], Maxent Variable Selection [42], or ENMeval [66], which offer rigorous processes of variable selection, can be useful in carrying out the selection of relevant variables.

Our test results of niche shifts can be explained by combined effects of environmental requirements (species ecology) and evolutionary changes allowing focal species to occupy newer areas and spread into novel environments. However, climatic data alone cannot accurately delineate shifts in fundamental or realized niches. A species range can expand as a result of local adaptations including gene flow and dispersal [67]. Moreover, frequent introductions from multiple sources could also produce novel genetic combinations through hybridization helping invasive to adapt in a new environment [3]. A genetically hybridized superior population can utilize new environmental conditions as compared to their parents [68]. Additionally, the founder effect could further help in rapid adaptations by expressing beneficial fitness-related alleles [69]. Moreover, areas in the invaded range that do not match the conditions in the native range could be interpreted as shifts due to evolution or adaptation. However, these may also represent landscapes which are part of the species’ native environments, but have not been documented due to their absence as a result of other factors such as competition, predation, host distribution, etc. Distinguishing these two patterns, to assign differences in niche occupancy between the native and invaded range implies based on a mechanism, seems difficult with this data. Additional data on species physiological thresholds from both native and introduced ranges must be taken into consideration when available.

The results show that all introduced WW populations have different environmental preferences and there may have been some evolution of the niche in the invasive ranges. The niche differences mirror the invasion history that was revealed through genetic analysis. The NA and Oceania populations originated primarily from Europe, the SA populations had multiple origins (including one that is still unknown), and the African populations had origins in SA, Oceania, and the same unknown source as part of the SA populations. The indicated range expansion in the SA and African populations may simply be an artifact due to the lack of sampling some unknown source population in Eurasia that was indicated by the genetic analyses. The unknown range may expand the niche in the native range once discovered and that may coincide with the apparent range expansions. It should, however, not be forgotten that the WW invasion is continuing and there are different strains of both the fungus and nematode as well as some parasitoids that all can affect the climatic niche and spread of the WW in invaded areas [70]. In addition, success of WW depends on the presence of a nematode that can sterilize female wasps, and the symbiotic fungus that the wasp larvae need to feed on, also travel with it.

The WW results also suggested that there was a considerable proportion of the introduced niche that was not yet invaded. This agrees with the results of modeling the potential range of the WW using CLIMEX [71]. There are areas of suitable native or exotic pines in Australia, Brazil, and North America that the WW could still disperse into. If portions of these additional areas differ in climate from the already occupied niche, then the invaded niche may be underestimated as was suggested for the SOD invaded niches.

ALB occupies a large climatic niche within its native range. This niche spans at least 24 degrees of latitude, and area that is characterized by significant temperature gradients [26]. The smaller niches estimated for ALB in the invaded regions are likely based on the small, localized invasive populations. Invasive ALB populations are subjected to intensive eradication programs [72], aimed at limiting the dispersal and spread into surrounding habitat. As such, the distribution of ALB is intentionally limited to these small infestations and the niches estimated for this species may not reflect the true invasive potential of this species.

Temperature was a dominant environmental factor driving niche differentiation between climatic niches within these non-native habitats speaks to the plasticity within the ALB populations. Invasive populations in North America and Europe occupy different niches, albeit niches that were encompassed, more or less, by the niche diversity within native range. ALB is known to be very plastic in its response to temperature [73], which suggests that new infestations may have the capacity to survive and establish in a range of habitats outside its native range. For example, ALB can survive in cool climates by altering the number of larval instars and requirements [73]. This ability to adapt may also explain the niche expansion beyond the borders of the native range seen in the EU populations. Although ALB in the introduced ranges currently seem to have filled their niche, their ability to adapt could expand the niche if new introductions occur. However, since the ALB in both NA and EU are under eradication that has limited the natural spread potential beyond the areas where the introductions occurred. This likely has limited the climatic niche, resulted in higher niche filling rates, and high niche stability as was observed in this study.

The large proportion of the SOD niche that is uninvaded in NA and Europe may indicate that the NA and EU populations are early in the invasion process and could disperse much more before reaching niche equilibrium. The SOD pathogen can disperse in two ways: via anthropogenic transport on plants and naturally via spores. The first means can lead to long-distance, even intercontinental transport. However, the spores naturally disperse slowly through wind driven rain with about half of the new infections occurring within 100 m of already infested trees [31]. Additionally, as the best set of climatic variables suggest, the pathogen has specific temperature and moisture requirements for infection that occur only seasonally and vary between years [74,75]. New infections also require susceptible hosts to be close to the infected trees. Most models of SOD spread and potential range in invaded areas include a susceptible host layer for this reason [76,77,78]. Adding a host variable to the niche model could improve the niche characterization. However, it has been shown that trying to model the niche for SOD when it is in an early stage of invasion will tend to underestimate the true niche so this may have affected our results [79]. The host range of this pathogen is still poorly understood and the recent host jump to Japanese larch was unexpected [80]. The results suggest niche evolution in the invasive ranges of SOD. This was also supported by a very high degree of niche expansion observed in both invasive populations of SOD and the fact that niche stability is very low (Table 4). This could result from the pathogen being introduced into areas with many new susceptible hosts and a less favorable or more variable climate so that rapid selection for the most viable fungal genotypes may have occurred. Another possibility exists, where the sampling of the native range is still limited, and thus may be underestimating the true extent of the native niche and therefore the overlap with the introduced niches. Although our study did not address subspecific niche adaptation, this could be done in the future. There are in fact three lineages of SOD in the Pacific Northwest, and British Columbia and some of the lineages diverged as long as 1 M years ago [30,81]. It is possible that there are two different areas that make up the NA niche 50% zone which could suggest that there may be different genotypes with different niches.

The lack of similarity between native and introduced ranges of DED shows that the populations in the introduced ranges have different environmental preferences and adaptations than those observed in the chosen native range. This could be caused by the presence of an entirely different species of the pathogen in Asia compared to the ones present in North America and Europe. The climatic niches in the invasive ranges of DED showed the highest degrees of niche filling compared to the native range possibly due to limited sampling of the pathogen in the presumed native range [82]. The niche expansion in North America and Europe could be linked to the differences in the fungal pathogen adaptation, the observed hybridization among the subpopulations of the pathogen and the availability of highly susceptible host species [33,37,83,84]. Brasier [83] has described the expansion of the Dutch elm disease outbreak (the second pandemic) following the appearance of a second species, *O. novo-ulmi*, that had optimum growth temperature (22 °C) lower than that of the original pathogen, *O. ulmi* (27.5–30 °C); it is likely that *O. novo-ulmi* is better adapted to more temperate and cooler climates than *O. ulmi,* which is considered to be better adapted to subtropical climates [33,85]. 

## 5. Conclusions

Most of our representative invasives were far away from reaching niche equilibrium and this finding was based completely upon observed species locations. We are aware of the fact that our results disregard the important biotic processes that may have delineated the species niche. In addition, limited samples from native and invasive ranges may have underestimated the actual distribution ranges of the invasives and hence the results obtained from this study should be assessed with caution. Additional observation data and information on physiological requirements of invasives derived from genomics from both native and introduced ranges would be useful to better understand and predict the behavior of the representative invasives in their respective newly introduced ranges. The information on climatic niche expansion and other important niche characteristics can prove to be a useful cost-effective tool in the decision-making on managing and monitoring representative invasives future spread and currently infested areas. This information can be used together with more spatial and temporal predictive models [86], which can predict the spread dynamics of invasive species in the infested range, and to design short and long-term management strategies of the invasives.

## Figures and Tables

**Table 1:** Climatic variables for niche analyses of selected forest invasives selected using the R package “Maxent Variable Selection” [42].

Variables	WW	ALB	SOD	DED	Variable Details
Bio1	*✓*	*✓*		*✓*	Annual Mean Temperature
Bio2	*✓*				Mean Diurnal Range
Bio3				*✓*	Isothermality
Bio4		*✓*	*✓*		Temperature Seasonality
Bio6		*✓*			Min Temperature of Coldest Month
Bio7	*✓*			*✓*	Annual Temperature Range
Bio11		*✓*		*✓*	Mean Temperature of Coldest Quarter
Bio13		*✓*			Precipitation of Wettest Month
Bio14	*✓*		*✓*	*✓*	Precipitation of Driest Month
Bio15	*✓*		*✓*	*✓*	Precipitation Seasonality
Bio19			*✓*	*✓*	Precipitation of Coldest Quarter

WW: Sirex woodwasp; ALB: Asian longhorned beetle; SOD: sudden oak death; DED: Dutch elm disease.

**Table 2:** Summary of *p* values for the niche test of *Sirex Woodwasp* between native range Eurasia (EUA) and invasive ranges Africa, Oceania, North America (NA), and South America (SA) using all and selected climatic variables.

Region	Variables	Overlap (D)	Similarity Test	Uninvaded	Expansion	Stability
Africa	All	0.02	0.515	0.87	0.47	0.53
	Selected	0.02	0.624	0.57	0.31	0.69
Oceania	All	0.24	0.267	0.54	0.26	0.74
	Selected	0.14	0.178	0.70	0.00	1.00
NA	All	0.31	0.475	0.49	0.00	1.00
	Selected	0.19	0.347	0.57	0.00	1.00
SA	All	0.04	0.356	0.85	0.63	0.37
	Selected	0.01	0.366	0.91	0.57	0.43

**Table 3:** Summary of *p* values for the niche test of Asian long-horned beetle between native range in Asia and invasive range in Europe (EU) and North America (NA) using all and the selected climatic variables. Significant *p* values (α = 0.05) are represented by *.

Region	Variables	Overlap (D)	Similarity Test	Uninvaded	Expansion	Stability
EU	All	0.01	0.752	0.97	0.00	1.00
	Selected	0.33	0.980	0.06	0.00	1.00
NA	All	0.01	0.960	0.98	0.00	1.00
	Selected	0.17	0.010 *	0.00	0.00	1.00

**Table 4:** Summary of *p* values for the niche test of sudden oak death between the presumed native range Indochina (Vietnam) and invasive ranges Europe (EU) and North America (NA) using all and selected climatic variables.

Region	Variables	Overlap (D)	Similarity Test	Uninvaded	Expansion	Stability
EU	All	0.357	0.168	0.044	0.234	0.766
	Selected	0.161	0.554	0.585	0.580	0.420
NA	All	0.262	0.188	0.567	0.552	0.448
	Selected	0.009	0.267	0.989	0.773	0.227

**Table 5:** Summary of *p* values for the niche test of Dutch elm disease between native range Asia and invasive ranges Europe (EU) and North America (NA) using all and selected climatic variables. Significant *p*-values (α = 0.05) are represented by *.

Region	Variables	Overlap (D)	Similarity Test	Uninvaded	Expansion	Stability
EU	All	0.69	0.238	0.00	0.26	0.61
	Selected	0.49	0.347	0.00	0.22	0.78
NA	All	0.57	0.030 *	0.00	0.39	0.74
	Selected	0.18	0.089	0.00	0.57	0.44

## Appendix A

**Table A1:** Summary of *p* values for the niche test of *Sirex Woodwasp* between invasive ranges Africa, Oceania, North America (NA) and South America (SA) and native range Eurasia (EUA) using all and selected climatic variables (2→1). Significant *p* values (α = 0.05) are represented by *.

Region	Variables	Overlap (D)	Similarity Test	Uninvaded	Expansion	Stability
Africa	All	0.02	0.079	0.47	0.87	0.13
	Selected	0.02	0.099	0.31	0.57	0.44
Oceania	All	0.24	0.050 *	0.26	0.54	0.46
	Selected	0.14	0.030 *	0.00	0.70	0.30
NA	All	0.31	0.020 *	0.00	0.49	0.51
	Selected	0.19	0.396	0.00	0.57	0.43
SA	All	0.04	0.089	0.63	0.85	0.15
	Selected	0.01	0.030 *	0.57	0.91	0.09

**Table A2:** Summary of *p* values for the niche test of Asian long-horned beetle between invasive range in Europe (EU) and North America (NA) and native range in Asia using all and the selected climatic variables (2→1). Significant *p* values (α = 0.05) are represented by *.

Region	Variables	Overlap (D)	Similarity Test	Uninvaded	Expansion	Stability
EU	All	0.01	0.040 *	0.00	0.97	0.03
	Selected	0.33	0.059	0.00	0.06	0.94
NA	All	0.01	0.040 *	0.00	0.98	0.02
	Selected	0.17	0.119	0.00	0.00	1.00

**Table A3:** Summary of *p* values for the niche test of Sudden oak death between the presumed native range Indochina (Vietnam) and invasive ranges Europe (EU) and North America (NA) using all and selected climatic variables. Bold values represent niche comparison values between native versus each introduced ranges (1→2) whereas unbolded values represent comparisons between introduced ranges versus native range (2→1). Significant *p*-values (α = 0.05) are represented by *.

Region	Variables	Overlap (D)	Similarity Test	Uninvaded	Expansion	Stability
EU	All	0.357	0.267	0.234	0.044	0.956
	Selected	0.161	0.861	0.580	0.585	0.415
NA	All	0.262	0.010 *	0.552	0.567	0.433
	Selected	0.009	0.950	0.773	0.989	0.011

**Table A4:** Summary of *p* values for the niche test of Dutch elm disease between invasive ranges Europe (EU) and North America (NA) and native range Asia using all and selected climatic variables (2→1). Significant *p* values (α = 0.05) are represented by *.

Region	Variables	Overlap (D)	Similarity Test	Uninvaded	Expansion	Stability
EU	All	0.69	0.238	0.26	0.00	1.00
	Selected	0.49	0.307	0.22	0.00	1.00
NA	All	0.57	0.010 *	0.39	0.00	1.00
	Selected	0.18	0.307	0.57	0.00	1.00
